# Development and validation of a machine learning model to predict prognosis in liver failure patients treated with non-bioartificial liver support system

**DOI:** 10.3389/fmed.2024.1368899

**Published:** 2024-03-13

**Authors:** Shi Shi, Yanfen Yang, Yuanli Liu, Rong Chen, XiaoXia Jia, Yutong Wang, Chunqing Deng

**Affiliations:** ^1^Department of Health Statistics, School of Public Health, Shanxi Medical University, Taiyuan, Shanxi, China; ^2^Department of Infectious Disease, First Hospital of Shanxi Medical University, Taiyuan, Shanxi, China; ^3^The 1st School of Clinical Medicine, Shanxi Medical University, Taiyuan, Shanxi, China; ^4^Department of Health Statistics, School of Medical Sciences, Shanxi Medical University, Taiyuan, Shanxi, China

**Keywords:** non-bioartificial liver support system, liver failure, random survival forests, multivariate cox regression, nomogram

## Abstract

**Background and objectives:**

The prognosis of liver failure treated with non-bioartificial liver support systems is poor. Detecting its risk factors and developing relevant prognostic models still represent the top priority to lower its death risk.

**Methods:**

All 215 patients with liver failure treated with non-bioartificial liver support system were retrospectively analyzed. Potential prognostic factors were investigated, and the Nomogram and the Random Survival Forests (RSF) models were constructed, respectively. Notably, we evaluated the performance of models and calculated the risk scores to divide patients into low-risk and high-risk groups.

**Results:**

In the training set, multifactorial Cox regression analysis showed that etiology, hepatic encephalopathy, total bilirubin, serum alkaline phosphatase, platelets, and MELD score were independent factors of short-term prognosis. The RSF model (AUC: 0.863, 0.792) performed better in prediction than the Nomogram model (AUC: 0.816, 0.756) and MELD (AUC: 0.658, 0.700) in the training and validation groups. On top of that, patients in the low-risk group had a significantly better prognosis than those in the high-risk group.

**Conclusion:**

We constructed the RSF model with etiology, hepatic encephalopathy, total bilirubin, serum alkaline phosphatase, platelets, and MELD score, which showed better prognostic power than the Nomogram model and MELD score and could help physicians make optimal treatment decisions.

## 1 Introduction

Liver failure (LF) is a seriously life-threatening hepatic syndrome associated with numerous serious complications characterized by organ failure and high clinical mortality (1, 2). There is no specific treatment for LF, and the current treatment consists of general management, artificial liver support system therapy, and liver transplantation. However, the efficacy of comprehensive management is relatively slow. Donor liver scarcity and high treatment costs have made liver transplantation (LT) impracticable for most patients (3, 4).

In the past few decades, artificial liver support system (ALSS) therapy has developed into a therapeutic option for LF because it is able to temporarily replace part of the functions of the failing liver, remove harmful substances from the body, stabilize the internal environment, and reduce the burden on the liver (5). Several studies found that the non-bioartificial liver support system (NBAL) could afford survival benefits for LF patients (6–8). However, one study (9) found the deterioration of liver failure in some patients even after more than 10 treatments. The prognosis has emerged as a clinical challenge. Comprehensively digesting its risk factors and thus accurately estimating its prognosis is all the more important.

Nowadays, various prognostic models, including the Chinese Group on the Study of Severe Hepatitis B-ACLF (COSSH ACLF) (10), the PALS model (11), and the APM model (12) were deemed suitable to predict the prognosis in patients with acute-on chronic liver failure (ACLF) treated using NBAL. Nevertheless, LF can present as acute liver failure (ALF), subacute liver failure (SALF), ACLF (an acute deterioration of known or unknown chronic liver disease), and chronic liver failure (CLF) (13, 14). Whether these models serve to evaluate the prognosis of LF patients with NBAL is uncertain. Besides that, most of the models have been constructed based on the Cox regression that should initially be satisfied with the assumptions.

Yet, Random Survival Forests (RSF), proposed by Ishwaran et al., has few limitations. RSF is a newly introduced forest ensemble learner to analyze right-censored survival data (15). Given the limited survival data, it sought a model that best represented the data. Moreover, there is the possibility to rank the importance of the variables, thus filtering out variables with greater significance. Now, RSF has successfully applied to the risk prediction of several diseases (16, 17). Accordingly, in the present study, we investigated the factors affecting short-term prognosis in patients with LF treated with NBAL therapy and applied the nomogram and RSF to develop clinical prediction models, respectively. We hope to find the optimal models which will help clinicians identify patients with LF at different risk levels and make treatment decisions, regardless of presentation.

## 2 Materials and methods

### 2.1 Study population

Two groups of patients were identified retrospectively: one for model establishment (training set) and the other for model validation (validation set). The training group included patients admitted to the Department of Infectious Diseases at the First Hospital of Shanxi Medical University between January 2014 and December 2021. The validation group included patients admitted to the Department of Infectious Diseases at the First Hospital of Shanxi Medical University between January 2022 and December 2022. The study protocol conforms to the ethical guidelines of the 1975 Declaration of Helsinki (6th revision, 2008) as reflected in *a priori* approval by the Ethics Committee of the First Hospital of Shanxi Medical University research committee, and the requirement for informed consent was waived owing to the retrospective nature of the study.

The inclusion criteria were as follows: (1) patients with SALF and CLF who were diagnosed according to the Guidelines for Diagnosis and Treatment of Liver Failure, and patients with ACLF and ALF were, respectively, diagnosed according to the consensus recommendations of the Asian Pacific Association for the Study of the Liver and the recommendations of the American College of Gastroenterology (13, 14, 18); (2) patients that underwent NBAL therapy; (3) age ranging from 18 to 75 years; (4) hospitalization time for at least 1 day; (5) complete availability inpatient clinical data, including basic patient information (name, gender, age, classification of LF, etiology, contact information and prior medical history), non-biological artificial liver treatments (number of treatments, mode of treatment and date of treatment), and clinical parameters (missing values <30%). The exclusion criteria included: (1) pregnancy or lactation; (2) HIV infection and other viral infections; (3) liver cancer and other malignancy; (4) any other severe extrahepatic chronic disease including severe renal, cardiac, respiratory, neurologic diseases, or other systemic diseases; (5) patients lacking timely follow-up.

**Figure 1:**
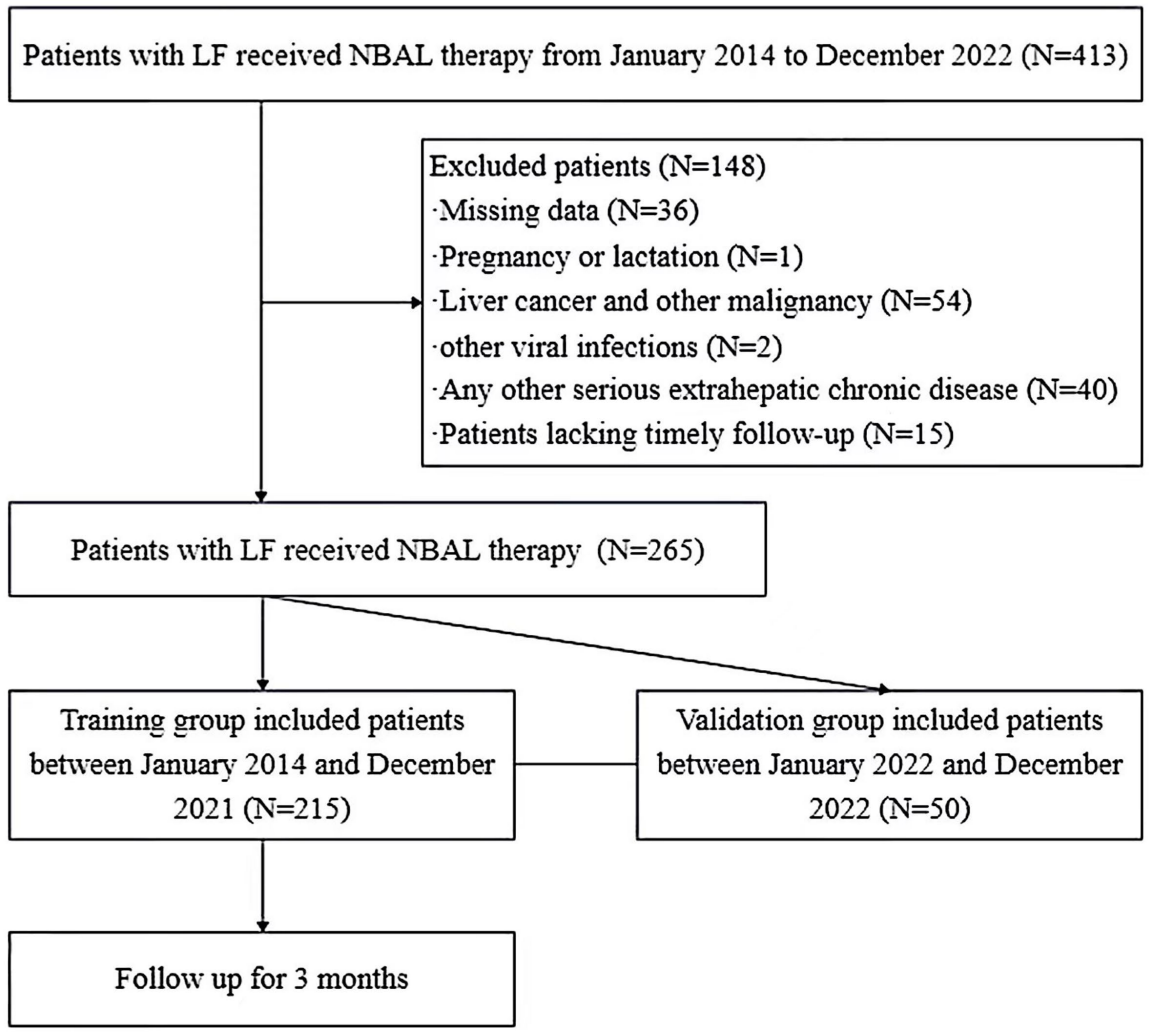
Flowchart of patient selection. LF, liver failure; NBAL therapy, non-bioartificial liver therapy.

All patients were followed up routinely at 3-month intervals from the first NBAL treatment with a cohort follow-up cut-off date of March 2023. The outcome of this study was all-cause mortality after 3 months of follow-up. During that time, if patients underwent liver transplantation, they were considered dead. The patient enrollment process is shown in Figure 1.

### 2.2 Treatment

All patients received standard medical therapy (SMT), including bed rest, adequate nutritional support, and correction of hypoproteinemia, water-electrolyte, and acid–base balance. Antiviral treatment was provided for HBV DNA-positive patients. Complications were also treated as follows: treatment of ascites included mainly etiologic therapy, sodium intake restriction, and diuretic therapy; treatment of hepatic encephalopathy included dietary restriction, lactulose, ammonia-lowering drugs, L-ornithine-L-aspartate (LOLA) and other supportive therapy.

The NBAL treatments in our study included plasma exchange (PE), double plasma molecular adsorption system (DPMAS), and DPMAS with sequential half-dose plasma exchange (DPMAS+PE). These methods are performed depending on the patient’s condition. PE applies to patients with other diseases in which macromolecules or pathogenic mediators bound to albumin are present in the blood, but PE alone is not recommended in patients with preexisting significant hepatic encephalopathy; DPMAS applies to patients with prehepatic failure, hyperbilirubinemia, and also in those with hepatic encephalopathy; DPMAS + PE is indicated in patients with hyperbilirubinemia, especially those with bilirubin levels >500 μmol/L (6, 19).

### 2.3 Data-collection

Clinical data collected included gender, age, main complications, etiology, classification of LF, number of treatments and treatment modality of NBAL, and laboratory parameters within 24 h before the first treatment, including alanine aminotransferase (ALT), aspartate aminotransferase (AST), albumin (ALB), alkaline phosphatase (ALP), total bilirubin (TBIL), cholinesterase (CHE), gamma-glutamyl transpeptidase (GGT), low-density lipoprotein (LDL), platelets (PLT), prothrombin time (PT), international normalized ratio (INR), creatinine (Cr), sodium, alpha-fetoprotein (AFP), and MELD score. Furthermore, the calculation of total bilirubin actual resident rate (TBARR), total bilirubin rebound rate (TBRR), and total bilirubin clearance rate (TBCR) was performed, which were calculated according to the following formula:

$$\begin{array}{l} \mathrm{MELD}=3.8\times \ln\left[\mathrm{TBIL}\left(\mathrm{mg}/\mathrm{dl}\right)\right]+11.2\times \ln INR+9.6\\ \times \ln\left[Cr\left(\mathrm{mg}/\mathrm{dl}\right)\right]+6.4\times \left(\begin{array}{l} \mathrm{aetiology}:0\;if\;\mathrm{cholestatic}\;\\ \mathrm{or}\;\mathrm{alcoholic},1\;\mathrm{otherwise} \end{array}\right) \end{array}$$

$$\mathrm{TBARR}=\left(\mathrm{postoperative}\;\mathrm{TBIL}\;\mathrm{at}\;48\;\mathrm{hours}\right)/\left(\mathrm{preoperative}\;\mathrm{TBIL}\right)$$

$$\mathrm{TBRR}=\frac{\begin{array}{l} \mathrm{postoperative}\;\mathrm{TBIL}\;\mathrm{at}\;48\;\mathrm{hours}-\\ \mathrm{immediately}\;\mathrm{postoperative}\;\mathrm{TBIL} \end{array}}{\mathrm{immediately}\;\mathrm{postoperative}\;\mathrm{TBIL}}$$

$$\mathrm{TBCR}=\frac{\mathrm{preoperative}\;\mathrm{TBIL}-\mathrm{immediately}\;\mathrm{postoperative}\;\mathrm{TBIL}}{\mathrm{preoperative}\;\mathrm{TBIL}}$$

### 2.4 Statistical analysis

Quantitative data were expressed as mean ± standard deviation (SD) when normally distributed and compared using Student’s *t*-test; when not normally distributed, they were indicated as median (interquartile range) and compared using the Mann–Whitney U test. Qualitative data were expressed as numbers (percentages), and chi-square tests were used for inter-group comparisons.

Baseline characteristics of the survival and death groups were compared using Student’s *t*-test, Mann–Whitney U-test, and Chi-square test. Potential predictors of prognosis were identified by univariate and multivariate analyses using Cox proportional risk regression models, using the forward stepwise method to screen for variables significantly associated with outcome. The test level α was set at 0.05, and the differences were considered significant at *p* < 0.05. The Nomogram and RSF models were developed, respectively, based on the above-mentioned characteristic variables. Bootstrap repeated sampling method (500 times) was used to perform internal validation of the prediction models. We compared the out-of-bag error rate and composite Brier score and plotted the prediction error curves. The optimal model was externally validated using calibration plots, decision-curse analysis (DCA) curves, and the area under the subject’s work characteristics curve.

All statistical analyses were performed using SPSS 25.0 software. Model construction was performed using R software version 4.2.1. Missing data were filled using the missForest package in the R software, version 4.2.1. The Nomogram model and calibration plots were constructed using the R package “rms”; the RSF model was constructed using the R package “randomForestSRC”; the DCA curves were drawn using the R package “dcurves”; “ROC curve analysis was performed using the R package “timeROC.”

## 3 Results

### 3.1 Patients characteristics

All 215 LF patients treated with NBAL participated in this study. Based on a three-month follow-up, patients were divided into survival (*N* = 138) and death groups (*N* = 77) in the training cohort. There were no significant differences in gender, classification of LF, treatment modality, and treatment frequency of NBAL in both groups (Table 1). A higher proportion of patients with hepatic encephalopathy (HE) was found in the death group than in the survival group (*p* < 0.05). Compared with the survival group, the levels of age, TBIL, PT, INR, Cr, MELD score, and TBARR were significantly higher in the death group (*p* < 0.05). Moreover, the levels of AST, ALP, CHE, GGT, LDL, and PLT in the death group were significantly lower than in the survival group (*p* < 0.05).

**Table 1:** Characteristics of survival and death groups in the training cohort.

	Survival group (*n* = 138)	Death group (*n* = 77)	*χ*^2^*/Z/t*	*p*-value
Gender (man/women)	83/55	52/25	1.154	0.306
Etiology					13.385	0.004
Hepatitis virus	48(34.8)	34(44.2)		
Medication	34(24.6)	8(10.4)		
Alcohol	28(20.3)	8(10.4)		
Others	28(20.3)	27(35.1)		
Classification					1.756	0.647
ALF	23(16.7)	14(18.2)		
SALF	13(9.4)	6(7.8)		
ACLF	88(63.8)	53(68.8)		
CLF	14(10.1)	4(5.2)		
Treatment modality				
PE	111(80.4)	64(83.1)	0.235	0.716
DPMAS	2(1.4)	1(1.3)	0.008	1.000
DPMAS+ PE	11(8.0)	3(39)	1.348	0.272
Main complications				
Ascites	89(64.5)	48(62.3)	0.099	0.769
HE	26(18.8)	33(42.9)	14.317	<0.001
Treatment frequency	2(2, 4)	2(1, 4)	−1.420	0.156
Age	47.36 ± 12.16	51.71 ± 11.64	2.225	0.011
ALT (U/L)	141.0(48.5, 416.3)	109.0(42.5, 351.0)	−1.007	0.314
AST (U/L)	209.5(90.0, 358.5)	131.0(81.0, 238.0)	−2.299	0.021
ALB (g/L)	30.22(27.70, 33.00)	30.00(28.20, 32.00)	−0.720	0.471
ALP (U/L)	122.7(106.0, 153.0)	109.5(95.0, 133.0)	−2.934	0.003
TBIL (μmol/L)	364.75 ± 152.75	436.76 ± 141.34	3.403	0.001
CHE (U/L)	1673.5(948.6, 2354.0)	1198.0(4.3, 1986.0)	−2.469	0.014
GGT (U/L)	83.14(62.00, 128.00)	72.72(44.00, 94.00)	−2.506	0.012
LDL (mmol/L)	1.10(0.93, 1.33)	0.97(0.84 ~ 1.22)	−2.490	0.013
PLT (×10^9^/L)	99.77(74.65, 131.56)	79.00(46.00, 115.77)	−3.034	0.002
PT (s)	25.60(21.99, 41.30)	29.79(25.00, 41.30)	2.982	0.003
INR	2.30(1.94, 2.91)	2.78(2.24, 3.61)	3.590	<0.001
Cr (μmol/L)	62.86(52.00 ~ 80.10)	75.00(55.00, 109.22)	2.301	0.021
Na (mmol/L)	135.22(132.14, 137.86)	134.00(131.00, 136.57)	−1.712	0.087
AFP	68.87(12.90, 168.15)	62.50(5.90, 140.24)	−0.977	0.328
MELD score	23.73(20.26, 26.98)	28.32(22.32, 33.16)	3.860	<0.001
TBARR (%)	78.19(67.37, 88.72)	85.06(76.93, 95.58)	3.338	0.001
TBRR (%)	24.46(2.10, 45.77)	32.42(11.04, 48.48)	1.128	0.259
TBCR (%)	37.94(28.97, 44.55)	21.99(35.67, 42.74)	−1.820	0.069

ALF, acute liver failure; SALF, subacute liver failure; ACLF, acute-on chronic liver failure; CLF, chronic liver failure; PE, plasma exchange; DPMAS, double plasma molecular adsorption system; DPMAS + PE, double plasma molecular adsorption system with sequential half-dose plasma exchange; HE, hepatic encephalopathy; ALT, alanine aminotransferase; AST, aspartate aminotransferase; ALB, albumin; ALP, alkaline phosphatase; TBIL, total bilirubin; CHE, cholinesterase; GGT, gamma-glutamyl transpeptidase; LDL, low-density lipoprotein; PLT, platelets; PT, prothrombin time; INR, international normalized ratio; Cr, creatinine; AFP, alpha-fetoprotein; MELD, Model for End-stage Liver Disease; TBARR, total bilirubin actual resident rate; TBRR, total bilirubin rebound rate; TBCR, total bilirubin clearance rate.

As shown in Table 2, we conducted the univariable Cox analysis on 25 potential factors, and the result determined 12 significant variables, including etiology, HE, age, ALP, TBIL, PLT, PT, INR, Cr, MELD score, TBARR, and TBCR. Additionally, the multivariable Cox regression analysis revealed that independent predictors of LF with NBAL treatments were etiology, HE, ALP, TBIL, PLT, and MELD scores.

**Table 2:** Results from univariate Cox regression and multivariate Cox regression.

	Univariate Cox regression						Multivariate Cox regression					
Variables	B	SE	Wald	*P*	HR	95%CI	B	SE	Wald	*P*	HR	95%CI
Gender	−0.213	0.243	0.768	0.381	0.808	0.501–1.302						
Etiology			12.853	0.005					12.162	0.007		
Etiology 2/Etiology 1	−0.908	0.393	5.334	0.021	0.403	0.187–0.872	−0.487	0.406	1.436	0.231	0.615	0.277–1.363
Etiology 3/Etiology 1	−0.746	0.393	3.605	0.058	0.474	0.219–1.024	−0.922	0.396	5.410	0.020	0.398	0.183–0.865
Etiology 4/Etiology 1	0.287	0.258	1.237	0.266	1.332	0.804–2.209	0.371	0.262	1.998	0.158	1.449	0.866–2.424
Classification			1.611	0.657								
Classification 2/Classification 1	−0.262	0.488	0.287	0.592	0.770	0.296–2.003						
Classification 3/Classification 1	−0.052	0.301	0.030	0.863	0.949	0.527–1.711						
Classification 4/Classification 1	−0.648	0.567	1.305	0.253	0.523	0.172–1.590						
Treatment modality												
PE	−0.121	0.304	0.157	0.692	0.886	0.488–1.609						
DPMAS	0.202	1.007	0.040	0.841	1.224	0.170–8.805						
DPMAS+ PE	0.604	0.589	1.051	0.305	1.829	0.577–5.803						
Main complications												
ascites	0.110	0.235	0.218	0.640	1.116	0.704–1.770						
HE	−0.886	0.231	14.759	<0.001	0.412	0.262–0.648	−1.048	0.242	18.791	<0.001	0.351	0.218–0.563
Treatment frequency	−0.055	0.073	0.568	0.451	0.946	0.820–1.092						
Age	0.025	0.010	6.569	0.010	1.025	1.006–1.045						
ALT (U/L)	0.000	0.000	1.349	0.246	1.000	0.999–1.000						
AST (U/L)	0.000	0.000	0.210	0.647	1.000	1.000–1.000						
ALB (g/L)	−0.019	0.026	0.555	0.456	0.981	0.932–1.032						
ALP (U/L)	−0.009	0.003	8.827	0.003	0.991	0.986–0.997	−0.008	0.003	8.406	0.004	0.992	0.987–0.997
TBIL (μmol/L)	0.003	0.001	12.361	<0.001	1.003	1.001–1.004	0.003	0.001	14.098	<0.001	1.003	1.001–1.005
CHE (U/L)	0.000	0.000	3.721	0.054	1.000	1.000–1.000						
GGT (U/L)	−0.003	0.002	3.745	0.053	0.997	0.993–1.000						
LDL (mmol/L)	−0.485	0.329	2.180	0.140	0.616	0.323–1.172						
PLT (×10^9^/L)	−0.008	0.003	9.351	0.002	0.992	0.987–0.997	−0.006	0.003	5.045	0.025	0.994	0.988–0.999
PT (s)	0.027	0.008	11.556	0.001	1.027	1.012–1.044						
INR	0.283	0.071	16.037	<0.001	1.327	1.156–1.525						
Cr (μmol/L)	0.002	0.001	4.315	0.038	1.002	1.000–1.004						
Na (mmol/L)	−0.031	0.024	1.582	0.208	0.970	0.925–1.017						
AFP	−0.001	0.001	0.797	0.372	0.999	0.997–1.001						
MELD score	0.075	0.013	31.930	<0.001	1.078	1.050–1.107	0.048	0.014	11.315	0.001	1.049	1.020–1.079
TBARR (%)	0.010	0.004	8.746	0.003	1.010	1.004–1.017						
TBRR (%)	0.002	0.003	0.270	0.603	1.002	0.995–1.009						
TBCR (%)	−0.011	0.004	6.966	0.008	0.989	0.982–0.997						

HR, hazard ration; CI, confidence interval; Etiology 1, hepatitis virus; Etiology 2, medication; Etiology 3, alcohol; Etiology 4, others; Classification 1, acute liver failure; Classification 2, subacute liver failure; Classification 3, acute-on chronic liver failure; Classification 4, chronic liver failure; PE, plasma exchange; DPMAS, double plasma molecular adsorption system; DPMAS + PE, double plasma molecular adsorption system with sequential half-dose plasma exchange; HE, hepatic encephalopathy; ALT, alanine aminotransferase; AST, aspartate aminotransferase; ALB, albumin; ALP, alkaline phosphatase; TBIL, total bilirubin; CHE, cholinesterase; GGT, gamma-glutamyl transpeptidase; LDL, low-density lipoprotein; PLT, platelets; PT, prothrombin time; INR, international normalized ratio; Cr, creatinine; AFP, alpha-fetoprotein; MELD, Model for End-stage Liver Disease; TBARR, total bilirubin actual resident rate; TBRR, total bilirubin rebound rate; TBCR, total bilirubin clearance rate.

### 3.2 Development of models in the training cohort

**Figure 2:**
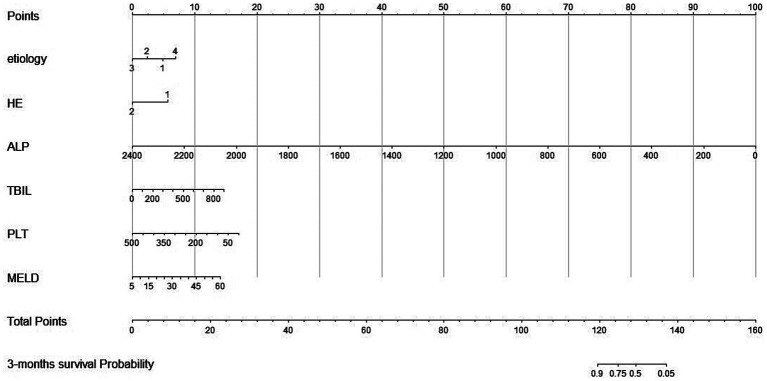
Nomogram for predicting 3-month overall survival for patients in the training cohort. Etiology 1, hepatitis virus; Etiology 2, medication; Etiology 3, alcohol; Etiology 4, others; HE, hepatic encephalopathy; ALP, alkaline phosphatase; TBIL, total bilirubin; PLT, platelets; PT, prothrombin time; MELD, Model for End-stage Liver Disease. Total points indicate the total points and are the sum of the six indicators.

According to independent predictors from multivariable Cox regression, we constructed a risk prediction nomogram model of LF with NBAL treatments (Figure 2).

The RSF model incorporates the above feature variables, and we trained the trees continuously based on mtry = 1 and ntree = 5,000 trees. Ntree = 2,000, which made the model stable, was selected as the optimal parameter (Supplementary Figures S1A). The optimal parameters, nodesize = 15 and mtry = 1, were determined by the grid search method (Supplementary Figures S1B). Based on the VIMP method, variables ranked in importance. All had VIMP values greater than 0, and the importance in descending order was MELD, HE, ALP, TBIL, PLT, and etiology (Supplementary Figures S2).

The internal validation results showed that both OBB and the composite Brier score of the RSF model (0.255, 0.161) are slightly lower than those (0.264, 0.169) of the Nomogram model. Meanwhile, the error curve of the RSF model was slightly lower than that of the Nomogram model as time increased, further indicating that the RSF model was relatively stable and reliable (Supplementary Figure S3).

### 3.3 Testing of models in the validation cohort

**Figure 3:**
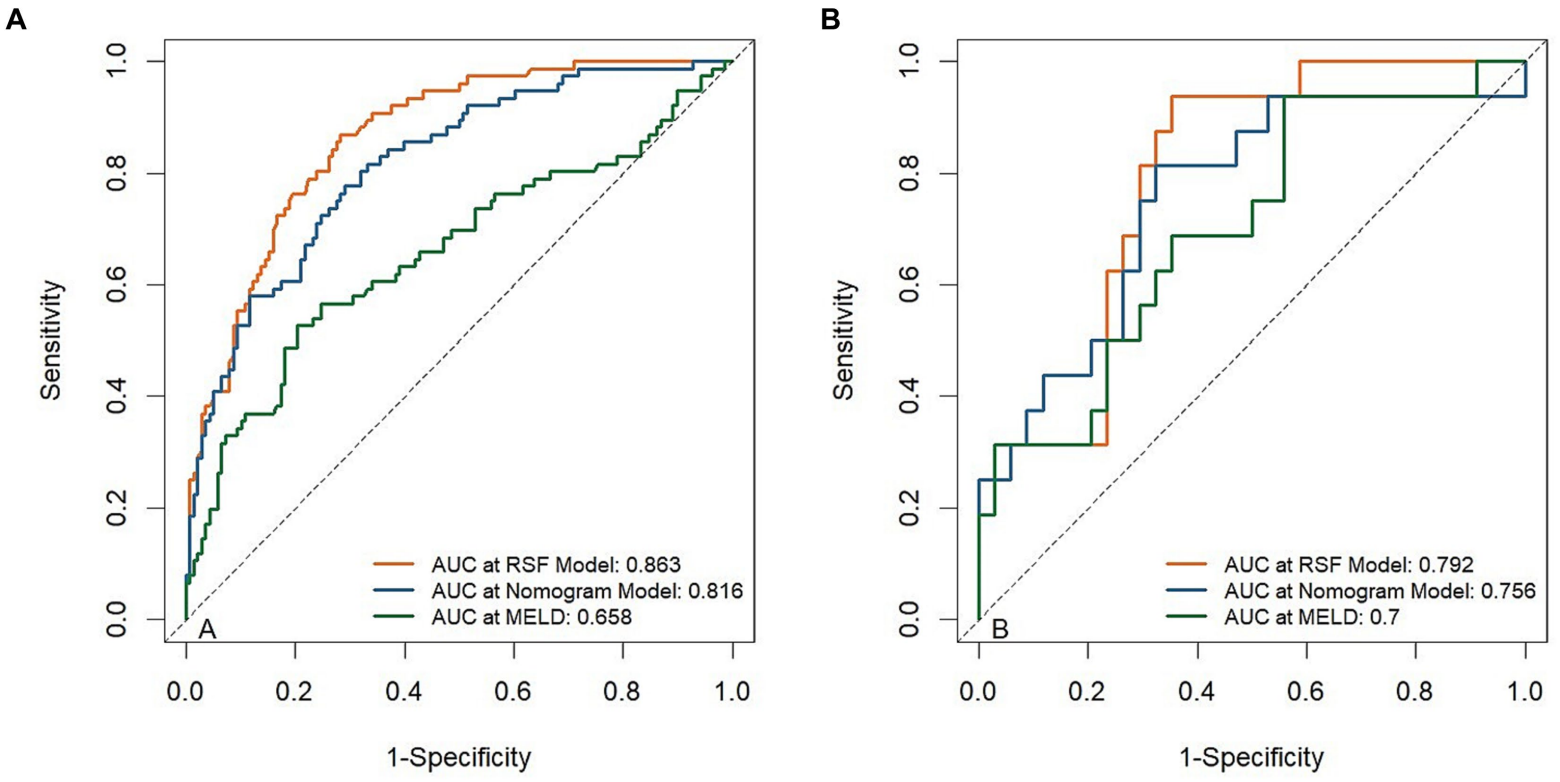
Receiver operating curves (ROC) for the abilities of risk models to predict 3-month mortality. ROC for risk models predicting 3-month mortality in the training cohort **(A)** and validation cohort **(B)**.

The developed model was further tested in the validation group. We plotted the ROC curves and calculated the corresponding AUC values in the training and validation cohorts. As shown in the ROC plots, there were significantly higher AUC values of the RSF model [0.863(95% CI, 0.815 ~ 0.912), 0.792(95% CI, 0.668 ~ 0.917)] in the training cohort, and validation cohort, compared to Nomogram model [0.816(95% CI, 0.758 ~ 0.874), 0.756(95% CI, 0.607 ~ 0.904)] and MELD [0.658(95% CI, 0.577 ~ 0.739), 0.700(95% CI, 0.545 ~ 0.855)] (Figure 3).

**Figure 4:**
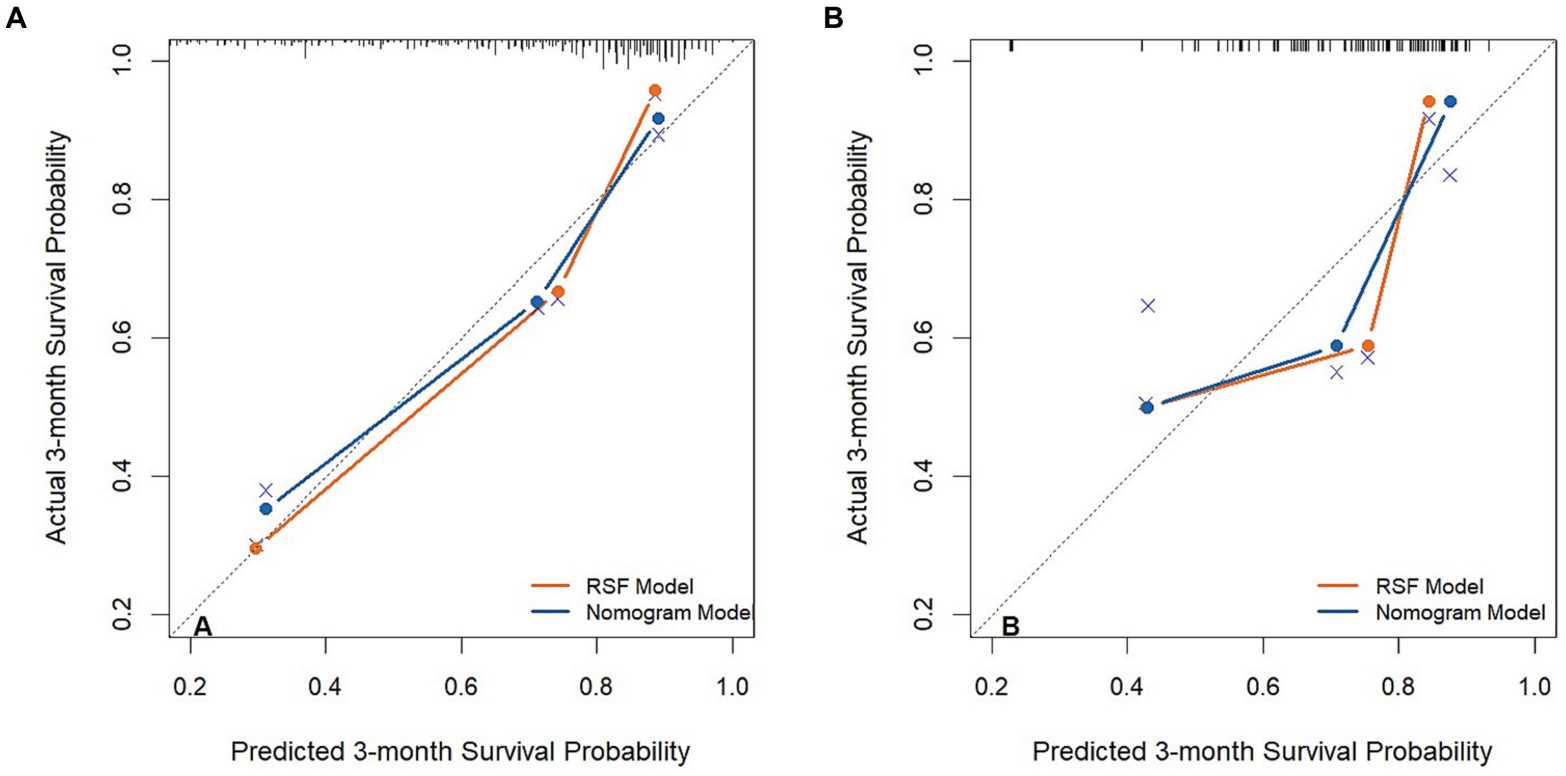
The calibration plot curves of models for predicting patients’ 3-month overall survival in the training cohort **(A)** and validation cohort **(B)**.

By observing the calibration plot both in the training cohort and the validation cohort, it is possible to roughly determine that there is a better agreement between the predicted survival probability and the actual observed results in the RSF model (Figure 4).

**Figure 5:**
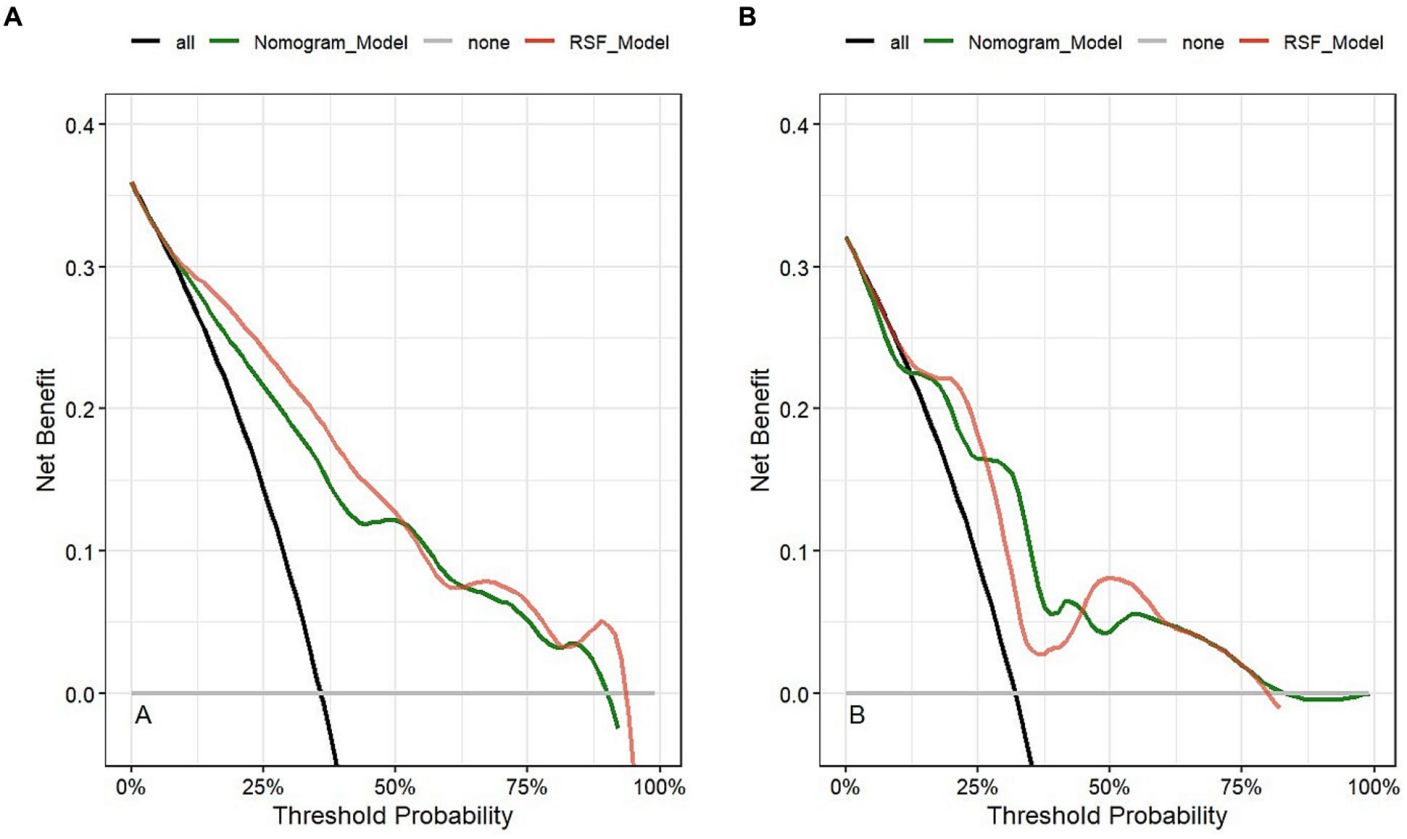
Clinical application evaluation of models in the training cohort **(A)** and validation cohort **(B)**.

Figure 5 is a decision curve depicting the clinical efficiency of the RSF model and the Nomogram model for predicting the prognosis of patients. Notably, the clinical efficiency of the RSF model was higher than that of the Nomogram model in the training cohort. However, the clinical efficiency of the RSF model was similar to that of the Nomogram model in the validation cohort. The net benefits of the RSF model were close to 0.94 and 0.81 in the training and validation cohorts, respectively.

Combining the results of OOB, the composite Brier score, ROC curve, calibration curve, and DCA curve, we found that the RSF model had better predictive value for the prognosis of LF patients treated with NBAL with high precision and clinical applicability.

### 3.4 Risk stratification of overall survival by the RSF model

The patients were divided into low-risk and high-risk groups by calculating the risk scores based on the RSF model. The optimal cutoff value for risk stratification of the RSF model was 11.97, which was determined using X-tile software version 3.6.1 (20). Supplementary Figure S4 shows the K-M survival curves for the different risk groups in the training and validation cohorts. As observed, patients in the low-risk group had a significantly better prognosis than those in the high-risk group (*p* < 0.001). Furthermore, as shown in Supplementary Tables S1, S2, the log-rank test results showed differences among groups.

## 4 Discussion

LF is a severely life-threatening clinical syndrome that has no effective treatment to improve patients’ clinical course. However, some LF patients with reversible potential may have a chance to be corrected by aggressive artificial liver therapy (21). PE has been widely used in China to remove toxic substances, but it requires a large amount of plasma and carries the potential risk of infection and allergy. Previous studies (22) have reported that PE helps to improve systemic inflammatory response syndrome (SIRS) and reduce the occurrence of multiple organ failure (MOF), thus prolonging the survival of patients. DPMAS fully utilizes plasma adsorption to remove inflammatory substances with plasma conservation and prevention of allergic reactions. These effects from DPMAS may help reduce TNF-α and IL-6 levels and effectively scavenge pro-inflammatory factors, with good survival benefits (23, 24). In recent years, it has been pointed out that DPMAS+PE combines both of these treatment modalities and has been considered as one of the best treatment modalities in case of plasma shortage and inability to perform liver transplantation (25, 26).

Although several previous prognostic models for LF have been applied and validated by some scholars, while specific models for predicting LF patients treated with ALSS have been published among Eastern and Western countries, few machine learning-based prognostic models have been published in detail for patients with LF treated with NBAL. We found that etiology, HE, ALP, TBIL, PLT, and MELD scores were associated with the outcome of LF patients treated with NBAL. Some scholars have reported that the prognosis of LF patients treated with NBAL is related to cirrhosis, TBIL, INR, infection, HE (11), Cr (7), age, MELD score (27), and AFP (12), which is generally consistent with our results. We also attempted to construct prognostic models based on the nomogram and RSF algorithm separately for LF patients treated with NBAL and select the better model while stratifying patients into different risk subgroups.

### 4.1 Etiology, complication, and prognosis

According to one study (28), approximately 1.32 million people worldwide died from alcohol-associated LF in 2017, making it the leading cause of liver-related mortality. Alcohol abuse increases intestinal permeability. In addition, lipids are peroxidized by reactive oxygen species produced by alcohol with its metabolites thus leading to hepatocellular damage (29, 30). Santhosh’s results (31) showed that low-volume plasma exchange and low-dose steroids improved survival in patients with alcohol-associated LF. We found that alcohol-associated LF was related to improved outcomes in patients treated with NBAL, although hepatitis virus-associated LF accounted for the highest proportion of LF. It suggests that more attention should be paid to this population of alcohol-associated LF. HE is a severe neuropsychiatric complication observed in patients with liver failure. The pathophysiology of HE is sophisticated and incompletely understood, but hyperammonemia and cerebral hemodynamic dysfunction appear to be central to the pathogenesis of HE (32). Some scholars have also hypothesized that HE may predict prognosis in patients with liver failure (33). Cai’s results (34) showed that HE, more commonly seen in LF patients presenting with infection, was an independent risk factor for 90-day mortality in ACLF patients showing infection. Du’s results (11) showed HE was the independent predictor of 3-month prognosis in patients with HBV-ACLF treated with PE therapy, which is generally consistent with our results.

### 4.2 Clinical indicators and prognosis

TBIL serves as an essential indicator of liver metabolic function and a prognostic indicator of liver failure (35). Severe hepatic impairment causes a decrease in bilirubin binding, allowing unconjugated bilirubin to accumulate in the blood. Meanwhile, biliary stasis leads to the accumulation of bile acids and conjugated bilirubin in the liver and systemic system (36). Studies have shown that high TBIL is associated with poor prognosis in patients with liver failure treated with non-bioartificial liver support systems, which is consistent with our findings (11). Our study also found lower ALP levels in the death group compared to the survival group. It suggests that cholestasis may develop in patients with a low risk of death, while high-risk patients have possible limitations of capillary bile duct function (37). Thrombocytopenia may be driven by viral infection, changes in portal pressure, and splenomegaly (38). Williamson and Chapman (39) found that ALP worked as one of the indicators to determine prognosis. Mu et al. (40) found that platelet counts had a high value in predicting short-term outcomes in patients with hepatitis E virus-related acute liver failure (HEV-ALF). Despite the different study populations, these findings seem to strengthen our results.

### 4.3 MELD score and prognosis

MELD is a recognized score for predicting survival in patients with end-stage liver disease, including TBIL, INR, Cr, and etiology (41). Che et al. (42) found a significant reduction in total bilirubin levels and MELD scores after NBAL treatment compared to pre-treatment. Chen’s results (43) showed that PE-based NBAL performed best in patients with 30 ~ 40 MELD scores. A meta-analysis by Li et al. (44) showed that patients treated with NBAL with lower-level MELD scores had a higher 28-day survival rate. In turn, our results found that MELD >32.12 had a higher risk of death. Moreover, our results showed that the MELD score within 24 h before the first NBAL treatment was an independent predictor of 3-month prognosis in LF patients treated with NBAL. Also, we found that both RSF and Nomogram models offered remarkable benefits compared to the MELD score. The TBIL level after artificial liver treatment will decrease to some extent, while it will rebound inevitably after a period of time. We sought to investigate the relationship between three indicators, TBCR, TBRR, and TBARR, and prognosis. Univariate Cox regression results suggest that TBARR and TBCR were probably relevant to prediction. However, data from retrospective studies were affected by various factors. Further confirmation of this conjecture is needed in the future.

### 4.4 Previous prognostic models

There are several nomogram models for predicting the prognosis of LF patients treated with NBAL. Ma et al. (10) conducted a retrospective study to assess the prognostic value of six models to predict the prognosis of patients with liver failure treated with artificial liver, and reported that the COSSH ACLF (AUC: 0.806, 95% confidence interval [CI]: 0.753 ~ 0.853) was more accurate in predicting the short-term prognosis of patients with ACLF treated with ALSS. Zhou et al. (27) used the number of comorbidities, age, MELD score, and artificial liver pattern to construct a model to predict the survival of patients with liver failure treated with artificial liver. PALS score (AUROC = 0.818) was established by Du et al. (11) to screen the subgroups (PALS score of 3–5 who received 1–2 sessions of ALSS therapy, PALS score of 6–9 who received ≥6 sessions of ALSS therapy) who could benefit from PE-centered ALSS therapy. Xie et al. (12) proposed a new APM model (AUC:0.790, 95% confidence interval [CI]: 0.740 ~ 0.834) including AFP levels to predict 28-day survival in patients with hepatitis B virus-related chronic plus acute liver failure treated with an artificial liver support system. To the best of our study, time-dependent AUC, calibration curve, and DCA all suggested that the RSF model had potential clinical application value. Also, we distinguished between high-risk and low-risk patients based on the risk score calculated from the RSF model. K-M plots and log-rank analysis showed significant differences between the two groups. Therefore, we should focus highly on patients with risk scores higher than 11.97. In addition, we wanted to try to compare the RSF model with existing prognostic models, but in this retrospective study, the data lacked relevant indicators. Further studies will have to be done in the future.

### 4.5 Limitations of study

There are several limitations of our study. The first one concerns that this is a single-center retrospective study and the issue of selection bias cannot be completely avoided. The second one lies in the small sample size of our data both in the training and validation sets. Because patients with acute liver failure, sub-acute liver failure, acute-on-chronic liver failure, and chronic liver failure have a diverse survival rate, sub-group analyses are required. However, the small sample size of our data will lead to an overfitting of the results. The last one is that our findings are based on the Chinese population and cannot be extrapolated to other countries. In the future, a prospective large sample of data shared by multiple medical centers may increase the reliability and generalizability of the prediction model.

In summary, the RSF model allows for predicting the short-term prognosis of patients with liver failure treated with non-bioartificial liver support systems, including etiology, HE, ALP, TBIL, PLT, and MELD scores. Stratifying the risk scores of the model enables the promotion of individualized treatment. This model needs to be further validated in the future.

## Data availability statement

The datasets presented in this article are not readily available because protecting patients' privacy and interests. Requests to access the datasets should be directed to SS, sshi_zi@163.com.

## Ethics statement

The studies involving humans were approved by the Ethics Committee of the First Hospital of Shanxi Medical University Research Committee. The studies were conducted in accordance with the local legislation and institutional requirements. Written informed consent for participation was not required from the participants or the participants’ legal guardians/next of kin because the retrospective nature of the study.

## Author contributions

SS: Data curation, Formal analysis, Investigation, Methodology, Writing – original draft, Writing – review & editing. YY: Investigation, Methodology, Visualization, Writing – original draft. YL: Investigation, Writing – review & editing. RC: Data curation, Formal analysis, Investigation, Writing – review & editing. XJ: Writing – review & editing. YW: Data curation, Formal analysis, Writing – review & editing. CD: Funding acquisition, Methodology, Project administration, Software, Writing – original draft, Writing – review & editing.
