# Supplementary material for: Development and validation of a machine learning model to predict prognosis in liver failure patients treated with non-bioartificial liver support system
Source: Front Med (Lausanne). 2024 Mar 13;11:1368899. doi: 10.3389/fmed.2024.1368899 (PMC10965676; doi:10.3389/fmed.2024.1368899)
Supplement: Supplementary file 1 [file Data_Sheet_1.docx]

Supplementary Material

1. **Supplementary Tables and Figures**

**1.1 Supplementary Tables**

**Table S1** Log-rank test of risk scores at the RSF model in the training cohort

|  | risk scores 1 (n=183) | risk scores 2 (n=32) | *χ^2^* | *P* |
| --- | --- | --- | --- | --- |
| Deaths | 49 (26.8%) | 28 (12.5%) | 96.621 | <0.001 |
| Survivals | 134 (73.2%) | 4 (27.3%) |  |  |

*Note*: risk scores 1, the low-risk group; risk scores 2, the high-risk group.

**Table S2** Log-rank test of risk scores at the RSF model in the validation cohort

|  | risk scores 1 (n=44) | risk scores 2 (n=6) | *χ^2^* | *P* |
| --- | --- | --- | --- | --- |
| Deaths | 11 (25.0%) | 5 (83.3%) | 20.426 | <0.001 |
| Survivals | 33 (75.0%) | 1 (16.7%) |  |  |

*Note*: risk scores 1, the low-risk group; risk scores 2, the high-risk group.

**1.2 Supplementary Figures**


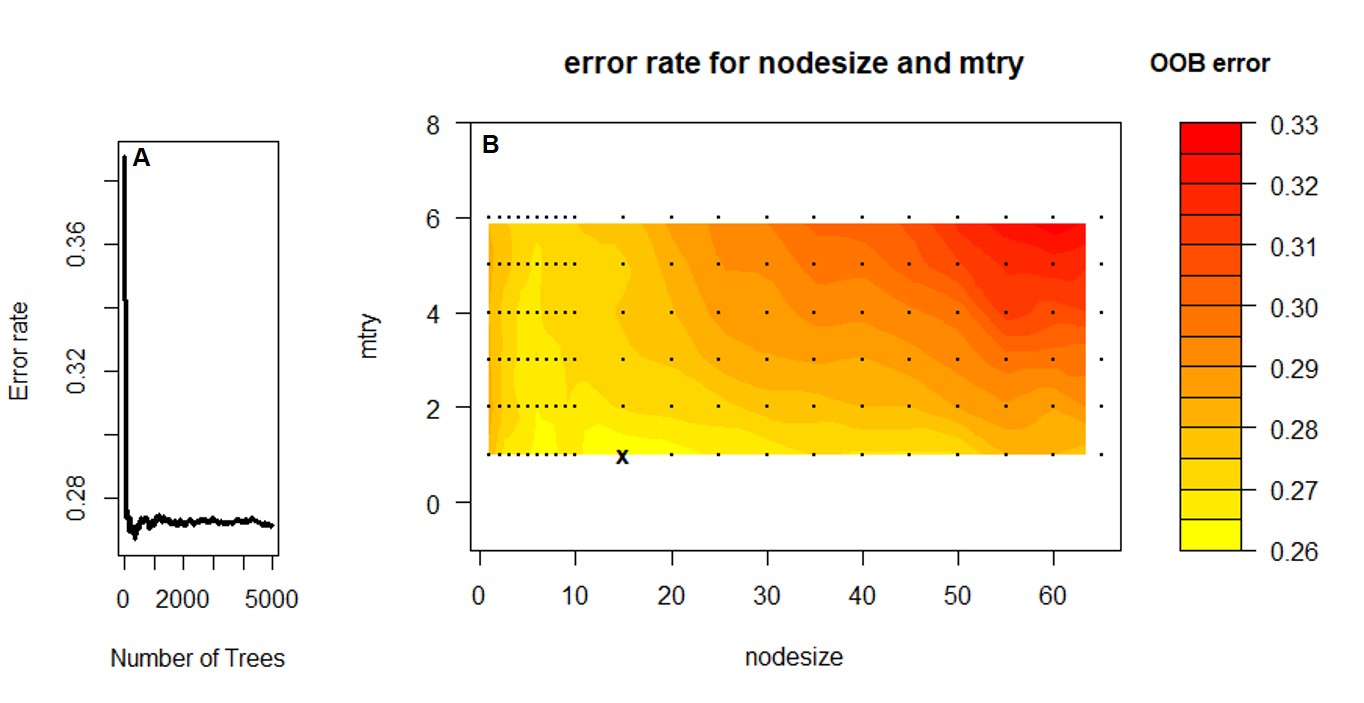


**Figure S1** Out-of-bag error rate plot (A) and tuning parameter plot (B) of RSF model. Number of trees is 2000 which makes the model stable. And the optimal parameters were nodesize=15 and mtry=1.


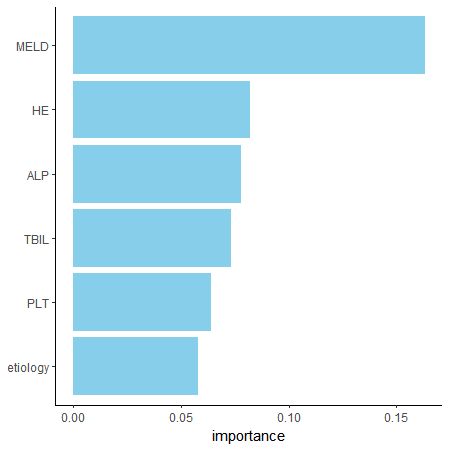


**Figure S2** VIMP values for each variable incorporated in the RSF model.


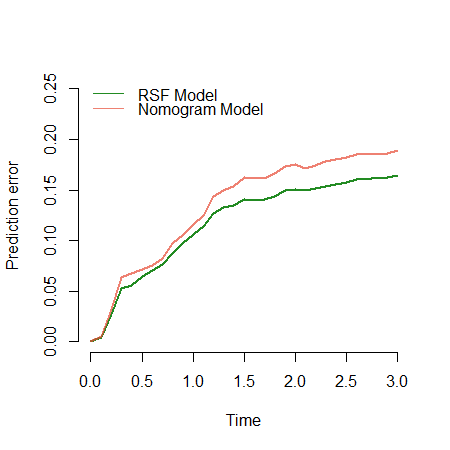


**Figure S3** Prediction error curves of the two models


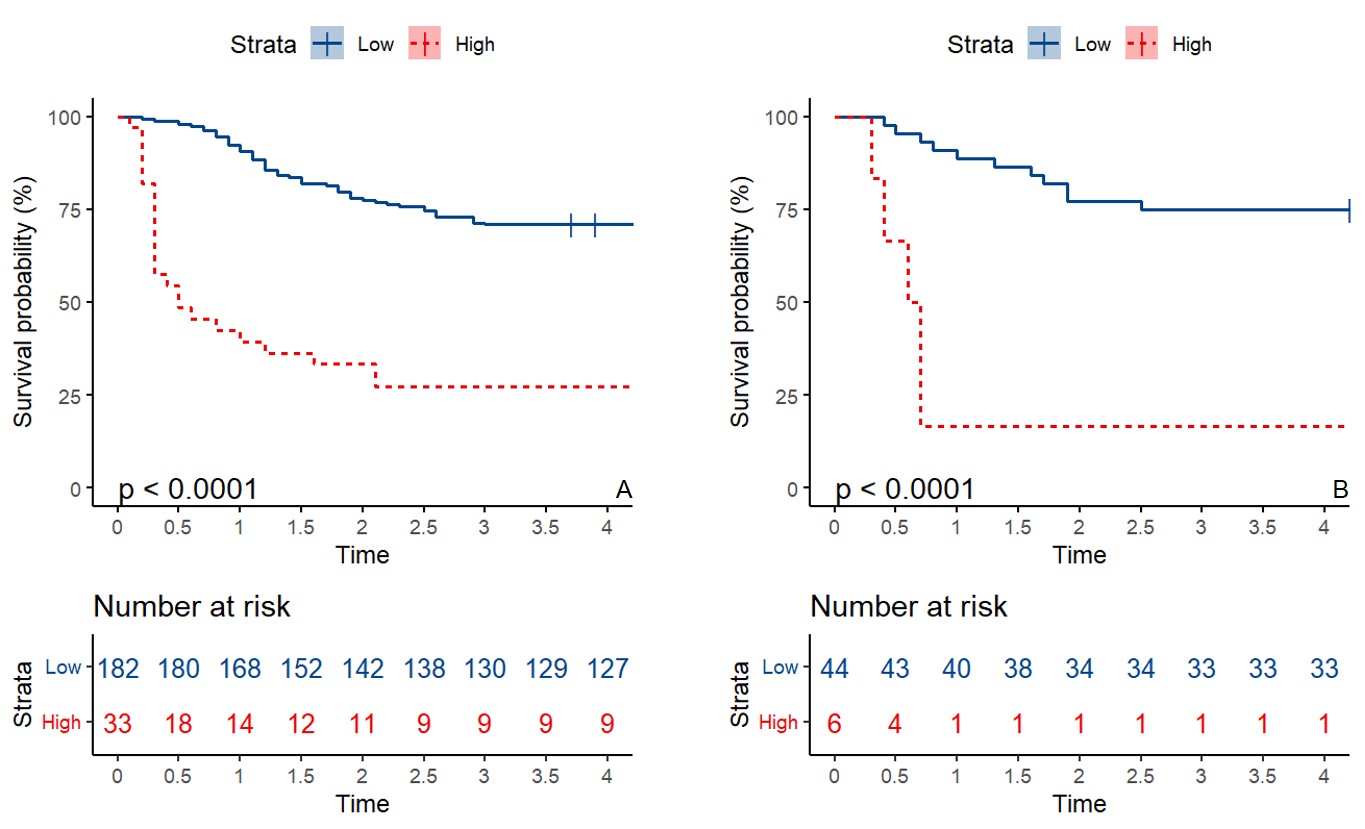


**Figure S4** Kaplan–Meier curve of risk scores at RSF model in the training (A) and validation (B) cohorts. The unit of time on the horizontal axis is month.
